# Effects of glycerol supply and specific growth rate on methanol-free production of CALB by *P. pastoris*: functional characterisation of a novel promoter

**DOI:** 10.1007/s00253-017-8123-x

**Published:** 2017-01-27

**Authors:** Verena Looser, Dominik Lüthy, Marcel Straumann, Katrin Hecht, Karel Melzoch, Karin Kovar

**Affiliations:** 1 Institute of Chemistry and Biotechnology, Zurich University of Applied Sciences (ZHAW), Campus Grüental, 8820 Wädenswil, Switzerland; 2 Department of Biotechnology, University of Chemistry and Technology Prague, Technická 5, 166 28 Prague 6, Czech Republic

**Keywords:** *Pichia pastoris*, *Candida antarctica* lipase B, Methanol-free, Specific productivity, Secretion, Product formation kinetics

## Abstract

As *Pichia pastoris* (syn. *Komagataella* sp.) yeast can secrete pure recombinant proteins at high rates, it is a desirable production system. The function of a novel synthetic variant of the AOX1 promoter was characterised comprehensively using a strain secreting *Candida antarctica* lipase B (CALB) as a model. A new time-saving approach was introduced to determine, in only one experiment, the hitherto unknown relationship between specific product formation rate (*q*_p_) and specific growth rate (*μ*). Tight control of recombinant protein formation was possible in the absence of methanol, while using glycerol as a sole carbon/energy source. CALB was not synthesised during batch cultivation in excess glycerol (>10 g l^−1^) and at a growth rate close to *μ*_max_ (0.15 h^−1^). Between 0.017 and 0.115 h^−1^ in glycerol-limited fedbatch cultures, basal levels of *q*_p_ > 0.4 mg g^−1^ h^−1^ CALB were reached, independent of the *μ* at which the culture grew. At *μ* > 0.04 h^−1^, an elevated *q*_p_ occurred temporarily during the first 20 h after changing to fedbatch mode and decreased thereafter to basal. In order to accelerate the determination of the *q*_p_(*μ*) relationship (kinetics of product formation), the entire *μ* range was covered in a single fedbatch experiment. By linearly increasing and decreasing glycerol addition rates, *μ* values were repeatedly shifted from 0.004 to 0.074 h^−1^ and vice versa. Changes in *q*_p_ were related to changes in *μ*. A rough estimation of *μ* range suitable for production was possible in a single fedbatch, thus significantly reducing the experimental input over previous approaches comprising several experiments.

## Introduction

*Pichia pastoris* (syn. *Komagataella* sp.) is an important host for the production and secretion of high levels of heterologous proteins. A variety of promoters are available, the most commonly used being the glyceraldehyde-3-phosphate dehydrogenase promoter (P_GAP_) for constitutive production and the alcohol oxidase 1 promoter (P_AOX1_) for inducible methanol-dependent production. Both systems have their drawbacks, particularly methanol as a substrate and P_GAP_ as a non-inducible promoter (Kovar et al. 2010; Mellitzer et al. 2014). Systems are sought in which production can be regulated without using methanol induction (Hartner et al. 2008; Prielhofer et al. 2013; Stadlmayr et al. 2010; Vogl and Glieder 2013; Vogl et al. 2016). Such carbon source-dependent promoters are either synthetic variants of the natural P_AOX1_ promoter, generated by selected (short) sequence deletions/insertions (Hartner et al. 2008), or newly identified natural carbon source-regulated promoters (Prielhofer et al. 2013; Stadlmayr et al. 2010; Vogl et al. 2016). For example, recombinant protein production can be repressed during batch phase with excess glycerol, and recombinant gene expression can be initiated during substrate-limited fedbatch cultivation with glucose (Prielhofer et al. 2013).

Regulation of synthetic P_AOX1_ variants is based on principles of repression/derepression, using glucose or glycerol as the only substrate (Capone et al. 2015; Mellitzer et al. 2014; Ruth et al. 2010). At high concentrations of glycerol or glucose, all P_AOX1_ variants were repressed but were derepressed by lower substrate availability (also described as being substrate limited) during fedbatch culture (Capone et al. 2015; Mellitzer et al. 2014; Ruth et al. 2010). For these new P_AOX1_ variants, little is known about their functionalities and possible control by process strategies, i.e. specific productivities in relation to pre-set specific growth rates. For instance, derepression of a P_AOX1_ variant (i.e. maximum product formation) was determined at a specific glycerol uptake rate of 0.054 g g^−1^ h^−1^, corresponding to a specific growth rate of 0.024 h^−1^ (Capone et al., 2015). However, for the widely applied *P. pastoris* system, in general, maximum recombinant product formation has been reported at various specific growth rates (Barrigon et al. 2015; Looser et al. 2015). The same promoter and substrate combination tended to have a greater influence on product formation kinetics than similar categories of recombinant products (Looser et al. 2015). Specific rates of P_GAP_-controlled protein production with glucose as a substrate typically increase with specific growth rates close to *μ*_max_ (Buchetics et al. 2011; Maurer et al. 2006; Rebnegger et al. 2014). The opposite is often found for P_AOX1_-controlled high-level recombinant protein formation induced by methanol, where maximum specific secretion rates were observed at a *μ* considerably below *μ*_max_ (Kobayashi et al. 2000; Potgieter et al. 2010; Wu et al. 2010). The main differences among the established production systems are the strain design (i.e. using an inducible or constitutive promoter) and the carbon/energy substrate (methanol or glucose). Maximum specific growth rate was much higher with glucose (*μ*_max_ ≤ 0.28 h^−1^ by Paulova et al. 2012) than with methanol; *μ*_max_ ≤ 0.14 or ≤0.035 h^−1^ for methanol utilisation slow phenotype (Mut^S^), respectively (Brierley et al. 1990). For this reason, the *μ* operational range for the design of a production process is different with different promoters.

*P. pastoris* strains with novel P_AOX1_ variant promoters, which are cultured with glycerol, are a combination of two systems with respect to promoter features and substrate. In general, product formation is not predictable a priori and has to be determined empirically for each combination of promoter and heterologous gene (Potgieter et al. 2010). Product formation kinetics *q*_p_(*μ*), the relationship between specific product formation rate (*q*_p_) and the specific growth rate (*μ*), reflects the equilibrium between various steps until the product is secreted. Maximum specific productivity for any given heterologous protein may be reached when the fluxes of produced, folded and secreted protein are balanced.

In order to design a production process, the *q*_p_(*μ*) relationship has to be established. It is the basis of several attempts at rational process design and optimisation (d’Anjou and Daugulis 2001; Kobayashi et al. 2000; Maurer et al. 2006; Ohya et al. 2005; Zhang et al. 2005). In general, either numerous time-consuming fedbatch processes (Hang et al. 2008; Zhang et al. 2005) or continuous cultivations (Curvers et al. 2001; Jungo et al. 2006; Khasa et al. 2007; Maurer et al. 2006) are performed at different pre-set *μ* values to establish this relationship. However, for recombinant protein production controlled by P_AOX1_ variants, little information is available. To speed up and rationalise the highly demanding task of biotechnological process development, in which each construct needs to be empirically characterised, efficient generic approaches are being sought. In this respect, the use of dynamic process conditions for rapid physiological strain characterisation has been described (Spadiut et al. 2013; Spadiut and Herwig 2014). Different dynamic fedbatch approaches presently being pursued could enable the entire production range of *q*_s_ (and *μ*) to be investigated in one single experiment (Capone et al. 2015; Spadiut et al. 2014; Zalai et al. 2012). The major advantage of the fedbatch technique is that the specific growth rate is controlled by the rate of substrate addition. The added substrate is immediately utilised, and cells can therefore only grow as fast as substrate is added. In theory, continuously increasing and decreasing the rate of substrate addition in fedbatch cultivation should enable a range of *μ* values to be analysed repeatedly, while the age of the cells and biomass concentrations increase steadily. Such a dynamic approach therefore allows the entire production range to be covered in a single experiment. Furthermore, testing of process conditions in a fedbatch environment may reveal phenomena in later production processes that continuous experiments would fail to predict (Zalai et al., 2012).

In this study, a *P. pastoris* strain secreting *Candida antarctica* lipase B (CALB), under the control of a novel synthetic P_AOX1_ variant, was characterised physiologically to demonstrate the functionality of this novel promoter. An approach to dynamically vary the specific growth rate within a single fedbatch experiment was compared to several single exponential fedbatch cultivations, which were performed at several different pre-set *μ* values to establish the desired *q*_p_ = *f*(*μ*) relationship. During all fedbatch cultivations, the P_AOX1_ variant was derepressed and a basal level of CALB was achieved independent of the specific growth rate applied, whereas *q*_p_ values higher than basal were dependent on production time, specific growth rate and substrate availability.

## Material and methods

### Strain

Clone 13-H2 used in this study was constructed and provided by VTU Technology GmbH (Raaba-Grambach, Austria). Two copies of a pPZ plasmid (Naatsaari et al. 2012) carrying the codon-optimised *C. antarctica* lipase B gene (*calB*) fused to the codon-optimised mating α-factor leader signal sequence of *Saccharomyces cerevisiae* for extracellular product secretion were integrated into the *AOX1* locus of *P. pastoris* CBS 7435 Mut^S^ strain. Gene expression was controlled by an AOX1 promoter variant (P_AOX1_ variant), which is a further variation of d6 promoter described by Hartner et al. (2008). In addition, a synthetic protein disulphide isomerase sequence (pPK plasmid, two copies, Naatsaari et al. 2012) was co-expressed under control of the similar promoter as CALB (without a recombinant signal sequence, located in endoplasmic reticulum). Stock cultures were conserved in 24% glycerol at −80 °C.

### Culture media

Culture media and inoculum were prepared as previously described by Hyka et al. (2010). All chemicals used in this study were of puriss grade p.a., purchased from Sigma-Aldrich part of Merck (formerly Fluka, Buchs, Switzerland), unless otherwise stated. Glycerol was purchased from Hänseler AG (Herisau, Switzerland). For pre-cultures, the buffered glycerol complex medium (BMGY) was used (Invitrogen 2002). It contained 10 g glycerol, 10 g yeast extract, 20 g peptone, 100 mM potassium phosphate buffer (pH 6.0), 13.4 g yeast nitrogen base without amino acids and 0.4 mg biotin l^−1^.

For both batch and fedbatch cultures, a defined mineral medium adapted from Hellwig et al. (2001) was used containing 0.17 g CaSO_4_·2H_2_O, 2.86 g K_2_SO_4_, 0.64 g KOH, 2.3 g MgSO_4_·7H_2_O, 0.2 g EDTA, 7.23 g H_3_PO_4_ and 0.1 ml of polypropylene glycol (PPG), which were all autoclaved, and 4.35 ml of filter-sterilised PTM1 solution l^−1^ and 0.87 mg of biotin l^−1^, which were added separately. The PTM1 stock solution (Invitrogen 2002) contained 5.0 ml of 69% H_2_SO_4_, 3.84 g CuSO_4_, 0.08 g NaI, 3.0 g MnSO_4_·H_2_O, 0.2 g Na_2_MoO_4_·2H_2_O, 0.02 g H_3_BO_3_, 0.92 g CoCl_2_·6H_2_O, 20.0 g ZnCl_2_ and 65.0 g FeSO_4_·7H_2_O l^−1^. Batch cultures were typically performed at ≤30 g of glycerol l^−1^. Feed solution for fedbatch cultures contained 588 g of glycerol, 2.4 mg of biotin and 12 ml of PTM1 solution kg^−1^.

### Bioreactor cultivations

Cultivations were performed either in a 14-l stirred tank bioreactor (Mavag, Neunkirch, Switzerland) at 18 l min^−1^ airflow (without any oxygen enrichment), 1400-rpm agitator speed and up to 0.5 bar overpressure; in an 18-l stirred tank bioreactor (Bilfinger Industrietechnik, Salzburg, Austria) at 18 l min^−1^ airflow (without any oxygen enrichment), 1000–1500-rpm agitator speed and up to 0.5 bar overpressure; or in a 50-l stirred tank bioreactor (Mavag, Neunkirch, Switzerland) at 57–65 l min^−1^ airflow (without any oxygen enrichment), 800-rpm agitator speed and without overpressure. As product secretion was found to be higher at 25 °C than at 30 °C, all production phases were carried out at 25 °C. Ammonia solution (12.5%) and phosphoric acid (8.5%) were used to maintain the pH at a constant value of 5.0.

The subsequent batch cultures comprised batches A and B. After an initial batch phase starting with a glycerol concentration of 30 g l^−1^, glycerol was repeatedly pulsed into the culture in order to keep the concentration constantly between 10 and 50 g l^−1^. For the second phase (B), the culture broth was completely removed and the residual biomass in the bioreactor was resupplied with fresh medium (∼1 g l^−1^ cell dry weight (CDW)).

Two different fedbatch culture regimes were used, depending on the specific growth rate. Fedbatch cultivations with a constant specific growth rate <0.02 h^−1^ comprised a phase of biomass growth in batch and fedbatch modes and a production phase in fedbatch mode. Cultivations with a constant specific growth rate >0.02 h^−1^, as well as the *μ* shift experiments, comprised only a phase of biomass growth in batch mode and a production phase in fedbatch mode. The relative partial pressure of oxygen (pO_2_) in the medium was not regulated, which resulted in a continuous decrease in the pO_2_ level during the batch culture. After a rapid increase in pO_2_ due to glycerol depletion, the addition of glycerol solution was initiated. Exponential addition rates of gram of feed per hour supporting a desired constant specific growth rate were calculated based on Eq. (1).

1 $$ F(t)={F}_0\cdot {e}^{\mu \cdot t} $$

2 $$ {F}_0=\left(\frac{\mu}{Y_{x/ s}}+{m}_{\mathrm{s}}\right)\cdot \frac{x_0\cdot {V}_0}{w_{\mathrm{in}}} $$

The value of initial feed rate *F*_0_ (Eq. (2)) is given by the desired (initial) specific growth rate (*μ*), maximum biomass to glycerol yield of 0.59 g g^−1^ (*Y*_*x*/*s*_), specific maintenance rate of 0.005 g glycerol per gram biomass per hour (*m*_s_), initial concentration of CDW in gram per litre (*x*_0_) multiplied by the initial volume in litre (*V*_0_) and the mass fraction of substrate in the feed solution of 0.58 g g^−1^ (*w*_in_). Exponential feed profiles for biomass growth phases (prior to the production phase) were calculated with a desired specific growth rate of 70% of *μ*_max_ (0.12 h^−1^) and with sufficient time to reach 60 to 70 g l^−1^ of cell dry weight.

### *μ* - Shift experiment in fedbatch culture

Based on growth parameters (*μ*_max_, *Y*_*x*/*s*_) determined during batch cultivation, the feed profile to determine the optimum specific growth rate (*μ*_opt_) for product formation was designed. By linearly increasing or decreasing glycerol feed addition rates, specific growth rates were dynamically shifted during the given time periods. Due to the availability of substrate per biomass data, linear feed profiles resulted in specific growth rates changing in a sigmoidal manner. The linear function of feed rate *F*(*t*) in gram feed solution per hour is given by Eq. (3).

3 $$ F(t)= a\cdot t+{F}_0 $$

The value of the initial feed rate, *F*_0_, is determined by the desired initial specific growth rate (*μ*) and is calculated as described by Eq. (2). A change in specific growth rate is determined by the value of the slope (*a*). This value was chosen according to the desired change in *μ*(*t*) (Eq. (6)). For this, the theoretical biomass production rate (*r*_*x*_) (Eq. (4)) was divided by the integrated *r*_*x*_ (Eq. (5)). For fedbatch planning by approximating *r*_*x*_(*t*), lower biomass-to-substrate yields at lower specific growth rates were not taken into account.

4 $$ {r}_x(t)= F(t)\cdot {w}_{\mathrm{in}}\cdot {Y}_{x/ s} $$

5 $$ x\cdot V(t)={x}_0\cdot {V}_0+{\int}_{t_0}^t{r}_x(t)\cdot dt $$

6 $$ \mu (t)=\frac{r_x(t)}{x\cdot V(t)} $$

A linearly increasing fedbatch profile was initiated after an adaptation time of several hours at a low constant rate. Time dependency of the cultivation, as well as cell density, was also considered in the design of the feed profile as *μ* ranges were studied repeatedly (in both increasing and decreasing *μ* directions). Both the duration of the experiment and the number of repetitions of phases with increasing and decreasing specific growth rates were technically limited by oxygen supply and maximum increase in volume. Only decreasing linear profiles were feasible at high biomass concentrations.

### Measurement of online and offline cultivation data

Measurements were performed as previously described by Hyka et al. (2010). The relative partial pressure of oxygen (pO_2_) in the medium, concentrations of both CO_2_ and O_2_ in the exhaust gas (extended process gas analyser; Secure Cell formerly Biospectra AG, Schlieren, Switzerland), pH, temperature, reactor overpressure and reactor weight were all monitored online. The biomass concentration was determined gravimetrically as CDW. Samples were centrifuged for 5 min at 13,000 rpm (5415R; Eppendorf, Hamburg, Germany) in pre-weighed 2-ml Eppendorf tubes that had been dried to a constant weight at 105 °C (Heraeus Instruments, Zurich, Switzerland). The concentrations of glycerol and other metabolites were determined by high-pressure liquid chromatography (HPLC) using an LC-20AB device equipped with autosampler SIL-20A, thermostated column oven CTO-20A and refractometer detector RID-10A (all produced by Shimadzu). The Aminex HPX-87H column, with an internal diameter (i.d.) of 7.8 mm (Bio-Rad, Munich, Germany), was run at 40 °C at a flow rate of 0.6 ml min^−1^ under isocratic conditions, with 2.5 mmol H_2_SO_4_ and an injection volume of 25 μl.

### Data analysis

#### Biomass concentration

Biomass concentration is given as gram cell dry weight per litre of culture broth (suspension). Product activity, concentration of total protein and glycerol concentration are stated per litre of supernatant. For calculation of derived values, all concentrations were multiplied by the corresponding volume (either total volume of broth or volume of supernatant).

#### Specific rates and yield coefficients

For cultivation phases with a constant specific growth rate, integral values for specific growth rate and yield coefficients were estimated by linear regression. Specific product formation rates were obtained by multiplication of product-to-biomass yields by the corresponding specific growth rate. The error bars indicate the 95% confidence interval estimated by linear regression. Time courses of specific conversion rates were calculated based on smoothed data using robust mode of Loess algorithm (Software Igor Pro; WaveMetrics, Lake Oswego, OR, USA). Each growth phase was smoothed separately, smoothed time courses being presented together with the raw data.

#### Calculation of biomass characteristics from online data

The time course of theoretical biomass productivity *r*_*x*_ in gram biomass was calculated by carbon balancing the online data available according to Eq. (7).

7 $$ {r}_x(t)=\left({r}_{\mathrm{s}}-{r}_{{\mathrm{CO}}_2}\right)\cdot {w}_x/ C $$

Carbon dioxide production rate, in gram carbon per hour (*r*_CO2_), was subtracted from the substrate take-up rate, in gram carbon per hour (*r*_s_). The resulting rate (in carbon h^−1^) was multiplied by the ratio of biomass to carbon of 2.11 g CDW per gram carbon (*w*_*x*/*c*_), which was derived from the elemental biomass composition of CH_1.665_N_0.134_O_0.602_S_0.0039_ determined by Carnicer et al. (2009) for a Fab expressing *P. pastoris* strain. To obtain the theoretical biomass, *r*_*x*_ was integrated over time (Eq. (5)), and biomass loss through sampling was considered. Theoretical biomass time courses were scaled to offline measured cell dry weight. For fedbatch phases, the measured CDW to theoretical (calculated) biomass was within 100 ± 15 %.

All figures were created with the Igor Pro software (WaveMetrics, Lake Oswego, OR, USA).

#### CALB activity

Hydrolytic activity of CALB was quantified using the spectrophotometric *p*-nitrophenylbutyrate (pNPB) assay (Morawski et al. 2000). In 96-well microtiter plates, 180 μl of substrate solution was added to 20 μl of diluted culture supernatant and shaken for 15 s before release of *p*-nitrophenolate was measured spectrophotometrically at 405 nm for 5 min at 25 °C (DTX 880, Beckman Coulter Fullerton, CA, USA). Due to autolysis of pNPB, substrate solution was freshly made for each measurement. For 10.1 ml of substrate solution containing 4.17 mM pNPB, 10 ml Tris/HCl buffer (pH 8, 300 mM) and 0.1 ml pNPB stock solution (468 mM in DMSO) were thoroughly mixed (emulsified) ultrasonically and used immediately. Sample supernatants were diluted on ice with Tris/HCl buffer (pH 8, 300 mM) to achieve an increase in extinction between 0.1 and 0.4 per minute (only sample measurements with a slope in this range were considered). Increase in extinction was registered over 5 min. A linear slope was ensured by only taking values up to a maximum of 2 into account. For determination of activity, the slope of the blank was subtracted from the sample slope before multiplication by the sample dilution factor. For *p*-nitrophenolate, a molar extinction coefficient of 9.595 ml μmol^−1^ cm^−1^ (405 nm, pH 8) was used. One unit is defined as the amount of enzyme that hydrolyzes 1 μmol of pNPB to *p*-nitrophenolate per minute at 25 °C and pH 8. Activity values are the mean of individually diluted triple determinations normalised to the internal standard by a normalising factor. The internal standard row (25, 20, 15, 10, 5, 2.5, 0 g l^−1^ lyophilised powder of commercially available CALB) was measured with each new microtiter plate, and the slope was aligned to the average slope of 2555 U per gram of lyophilised commercially available CALB (c-Lecta, Leipzig, Germany, containing 10–20% protein according to information supplied by the manufacturer).

#### Protein concentration

Protein concentrations in the culture supernatant were determined by the Bradford method using the Protein Quick Start™ Bradford Protein Assay (Bio-Rad, Hercules, CA, USA). This assay was performed according to the manufacturer’s instructions, based on a specific microplate protocol and with bovine gamma-globulin as the relative protein standard.

The fraction of CALB in relation to total protein secreted into the supernatant was determined by microfluidic capillary electrophoresis (LabChip® 90 Electrophoresis System, PerkinElmer formerly Caliper Life Sciences, Waltham, MA, USA).

## Results

The P_AOX1_ variant is a novel promoter for use with *P. pastoris* to produce heterologous proteins that can be controlled without induction by methanol. Using a strain secreting CALB, the relationship between product formation and biomass growth, as well as the effect of external glycerol concentration, on product formation was elucidated. Furthermore, an efficient method to estimate the appropriate range of specific growth rates over which product formation is optimal was designed.

### Effect of biomass growth with excess glycerol on product formation

**Fig. 1:**
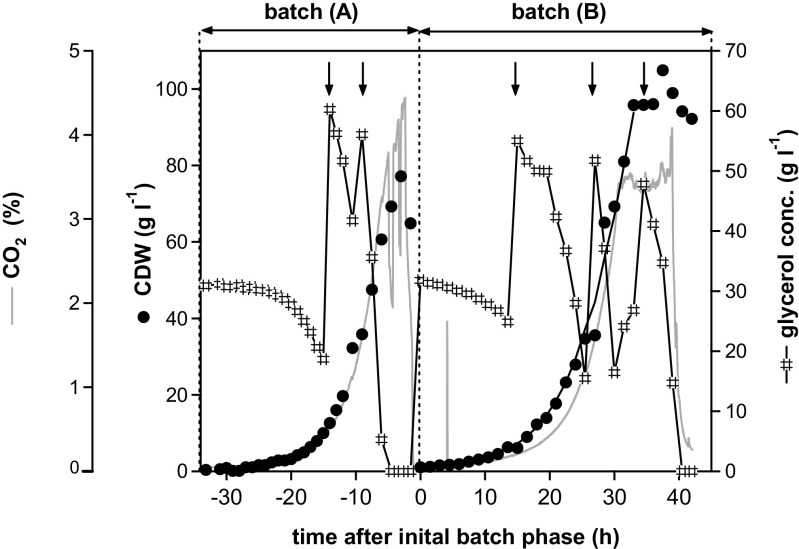
Batch cultivation with glycerol in excess. Cultivation comprised two consecutive batch cultures (phases A and B) with several pulses of glycerol, as indicated by *arrows*. The initial batch (**a**) was performed at 30 °C. After finishing this batch, a significant amount of the culture (at about 1 g l^−1^) was removed and the volume was replaced with fresh medium to start batch B at 1 g l^−1^ CDW and 25 °C (production temperature). Unlimited, exponential biomass growth with excess glycerol during batch phase B is indicated by simulated time courses of biomass concentration, given as *lines*. Time courses of the cell dry weight (CDW, biomass concentration) were simulated according to the phases and values stated in Table 1. Neither CALB activity nor protein concentration is shown, since under these conditions, no recombinant product was secreted

CALB production under the control of the P_AOX1_ variant was investigated in batch cultivations with glycerol in excess (10 to 50 g l^−1^). Excess of glycerol was maintained during two subsequent batch cultures (batch A and B) by glycerol pulses (Fig. 1). Batch A was performed analogous to batch-fedbatch experiments described later. Throughout the second batch phase (B), carbon dioxide concentration in the off gas constantly increased up to 40 h, indicating that glycerol was always in excess. Under these conditions, cells grew at 25 °C with a maximum specific growth rate of 0.15 ± 0.01 h^−1^ and a biomass to glycerol yield of 1.52 ± 0.15 g biomass per gram of carbon (Table 1). During batch cultivation with glycerol in excess, no product was synthesised since CALB activity was not detected in the supernatant or intracellularly (data not shown).

**Table 1:** Characteristics of growth and product formation

Related symbol				Exponential growth			Stable product secretion			
				*μ* (h^−1^)	Time^b^ (h)	*n* (−)	*q*_p_ (mg g^−1^ h^−1^)	*Y*_*p*/*x*_ (mg g^−1^)	Time^b^ (h)	*n* (−)
Figure 3	▲	Fedbatch 1		0.017 ± 0.001	22–68	16	0.43 ± 0.04^c^	25.5 ± 2.0	30–68	14
	♦	Fedbatch 2		0.038 ± 0.001	0–71	15	0.93 ± 0.06	24.6 ± 1.3	8–61	13
	●	Fedbatch 3	Phase 1	0.049 ± 0.001	0–41	22	1.15 ± 0.15	23.7 ± 3.0	5–19	8
			Phase 2				0.40 ± 0.06^c^	8.3 ± 1.3	19–39	11
Figure 2	■	Fedbatch 4	Phase 1	0.067 ± 0.003	2–40	21	1.03 ± 0.14	15.3 ± 2.0	6–23	10
			Phase 2				0.44 ± 0.03^c^	6.5 ± 0.4	23–38	8
	○	Fedbatch 5		0.094 ± 0.002	0–25	12	0.58 ± 0.05^c^	6.1 ± 0.5	11–25	11
	◊	Fedbatch 6		0.115 ± 0.004	0–25	12	0.48 ± 0.07^c^	4.2 ± 0.6	11–25	11
Figure 1	Δ	Batch *μ*_max_^a^		0.15 ± 0.005	6–33	19	ND	ND	6–33	19

For different fedbatch cultivations with exponential glycerol addition at 25 °C, phases of both constant specific growth rate (*μ*) and production (*q*_p_) were identified. Values for stable product secretion are based on total protein (secreted) which correlates with enzyme activity. Errors of calculated values are shown as 95% confidence intervals

*ND* not detectable, *n* number of measurement values

^a^Repeated batch cultivation phase B

^b^Time starting after initial batch phase

^c^Basal level of *q*_p_ 0.4 to 0.6 mg g^−1^ h^−1^ being independent of *μ*

### Product formation in fedbatch culture under glycerol-limited growth

**Fig. 2:**
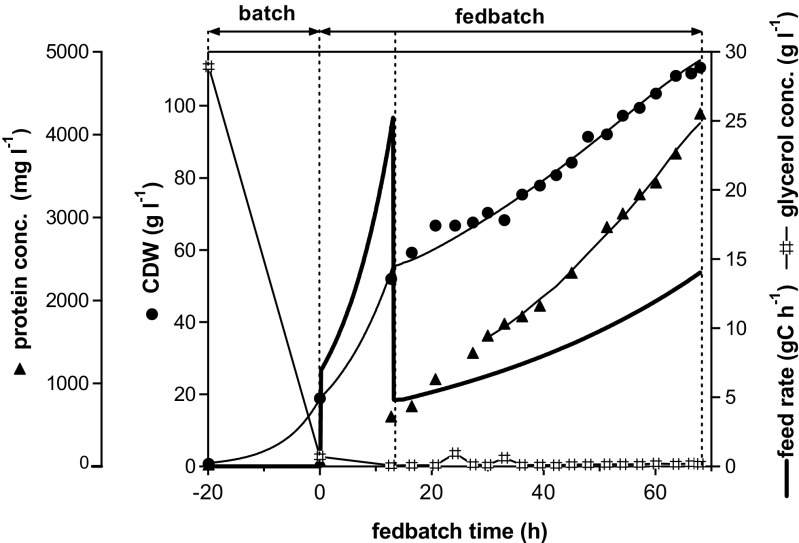
Performance of the synthetic P_AOX1_ variant in fedbatch culture. Determined concentrations are shown as *symbols*, and simulated time courses are given as *lines*. Cultivation comprised the following phases: *batch﻿:* unlimited, exponential biomass growth at 25 °C with excess glycerol resulted in a maximum specific growth rate of 0.15 h^−1^; formation of active CALB enzyme was repressed *fedbatch:* carbon-limited biomass growth with exponential addition of glycerol (feed rate shown by *thick line*) to support average specific growth rates of 0.09 h^−1^ (first fedbatch phase mainly to increase biomass) and 0.017 h^−1^ (second fedbatch phase mainly to build product), with production of active enzyme during both fedbatch phases. The phase of constant product formation (*q*_p_) is indicated by a *thin line*, corresponding to the time course of product concentration simulated accordingly to phases and values stated in Table 1 (fedbatch 1)

The potential of the synthetic promoter to control recombinant product formation was thought to depend on glycerol availability. The cultivation experiment to demonstrate control of product formation consisted of an initial batch phase followed by two fedbatch phases with two different specific growth rates (Fig. 2). Glycerol added during the fedbatch cultivation was immediately used down to <1 g l^−1^, and thus, specific growth rates were controlled by the specific addition rate of substrate (*q*_s_).

Most of the biomass was produced during the initial batch and the first fedbatch phases. The focus of the second fedbatch was on product formation. Production was initiated during the first fedbatch phase. After the second fedbatch phase, at 68 h, 4.2 g l^−1^ CALB was measured in the culture supernatant (Fig. 2), corresponding to an activity of 83,000 U l^−1^.

Product formation under the P_AOX1_ variant promoter was controlled by substrate (glycerol) availability. By changing between glycerol excess and glycerol limitation, product formation could be switched on and off without using methanol as an inducer.

### *μ* - Dependency of recombinant product formation

**Fig. 3:**
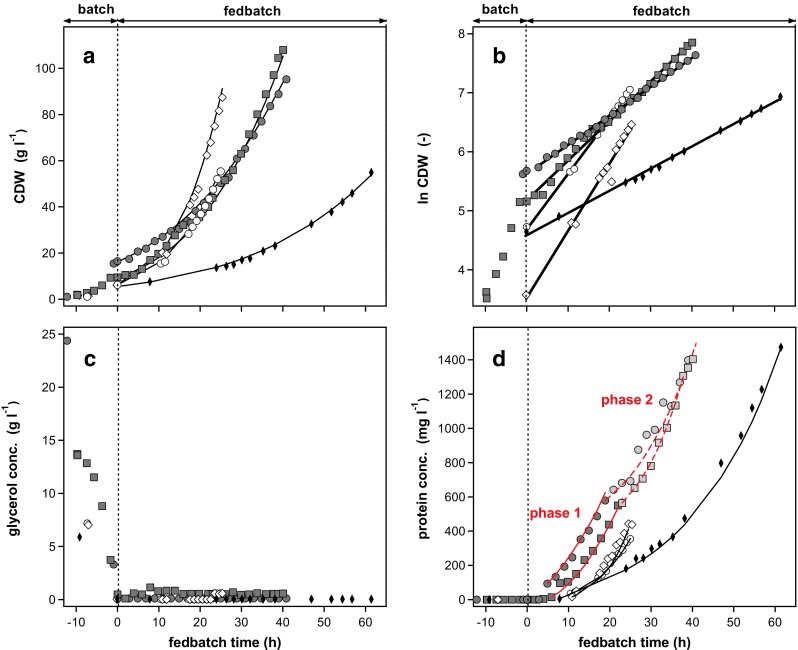
Fedbatch cultivations at different pre-set *μ* values, due to exponential feed addition.* Symbols* and corresponding cultivations are defined in Table 1. Constant specific growth rates ranging from 0.038 to 0.115 h^−1^ were supported during five individual fedbatch cultivations, each with exponential addition of glycerol. Phases of constant growth (*μ*) and product formation (*q*_p_) are indicated by simulated time courses of biomass concentration (graph **a**) and product concentration (graph **d**) given as *lines*. Time courses were simulated according to the phases and determined values as stated in Table 1. *Lines* in graph **b** represent linear regressions, whereas the slope corresponds to a constant specific growth rate

The relationship between specific rate of formation of the secreted product (CALB) and specific growth rate was investigated in a conceptual attempt with several exponential fedbatch cultivations at different pre-set *μ* values and with glycerol as the sole carbon and energy source (Fig. 3, Table 1).

After an initial batch phase with excess of glycerol, feed solution was added at an exponentially increasing rate. In all fedbatch cultivations, the continuously added glycerol was utilised immediately and the residual glycerol concentration was ≤1 g l^−1^ (Fig. 3c), driving specific growth rates pre-set at a constant value during each cultivation. Values of *μ* from 0.017 to 0.115 h^−1^ were investigated. The phases of exponential growth (with constant *μ*) were identified by linear regression of the biomass data (Fig. 3a, b, values given in Table 1). For all *μ* values tested, product concentration in the supernatant steadily increased between 5 and 11 h after the start of a fedbatch cultivation (Fig. 3d). Phases of stable product secretion were identified by determination of average specific secretion rates (*q*_p_), i.e. the amount of product secreted per gram of biomass per hour (Table 1).

Product secretion at a low specific growth rate of 0.038 ± 0.001 h^−1^ was stable for 53 h (Table 1, fedbatch 2). For fedbatch cultivations with higher specific growth rates, *q*_p_ values were not stable over the whole production phase and two phases of constant *q*_p_ were distinguished (Fig. 3d, Table 1). Cell growth of 0.067 ± 0.003 h^−1^ resulted in a constant specific product formation of 1.03 ± 0.14 mg g^−1^ h^−1^ for the first 23 h (phase 1, corresponding to 19.1 U g^−1^ h^−1^). Afterwards, product formation was reduced by 57% to a constant level of 0.44 ± 0.03 mg g^−1^ h^−1^ (phase 2). A comparable effect was found for a *μ* of 0.049 ± 0.001 h^−1^ (Table 1, fedbatch 3). This change in production performance cannot be attributed to a change in growth since the specific growth rate (determined by CDW) was constant during the whole fedbatch phase (Fig. 3b).

**Fig. 4:**
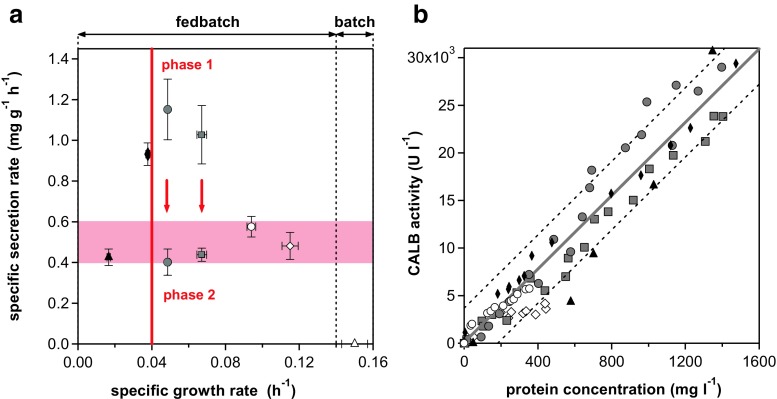
Recombinant product formation in relation to specific growth rate. *Symbols* and corresponding cultivations are defined in Table 1. **a** CALB secretion kinetics. Each *symbol* in this relationship represents the average value gained from an individual cultivation, either established during glycerol-limited cultivation (fedbatch) or excess of glycerol (batch, *open triangle* at 0.15 h^−1^ as maximum specific growth rate of 0.15 h^−1^). According to the established *q*_p_(*μ*) relationship, the optimum production range is ≤0.04 h^−1^ (achieved at ≤27% of maximum specific growth rate) and is indicated by a *vertical line*. The shift from initial high product formation (phase 1) followed by a lower but also stable second phase (phase 2) is indicated by *arrows*. Basal specific secretion rates of 0.4 to 0.6 mg g^−1^ h^−1^ are indicated by the *shaded area*. *Error bars* indicate the 95% confidence interval. **b** CALB activity in the supernatant is given as a function of protein concentration in the culture supernatant. Product activity was measured as hydrolytic activity (pNPB) in a spectrophotometric assay, and protein concentration was determined by the Bradford method. The linear slope is equivalent to a specific CALB activity of 19.3 ± 0.8 U mg^−1^. Errors are expressed as 95% confidence intervals (not visible in the given scale,) and prediction bands are shown as *dashed lines*

Based on these data, kinetics of CALB formation *q*_p_(*μ*) with glycerol as the sole carbon and energy source was established (Fig. 4a). Each symbol in this relationship represents the average value gained from individual fedbatch cultivations (Fig. 3, Table 1). This relationship revealed a high, stable product secretion of 0.93 ± 0.06 mg g^−1^ h^−1^ for a specific growth rate of 0.038 h^−1^ (Fig. 4). For fedbatch cultivations with a *μ* of 0.049 and 0.067 h^−1^, two different production phases were determined. Initially, high but transient secretion rates of 1.03 to 1.15 mg g^−1^ h^−1^ were observed, comparable to stable and still considerably high, secretion rates found when cells were grown at *μ* = 0.038 h^−1^. Interestingly, a basal level of *q*_p_ values between 0.4 and 0.6 mg g^−1^ h^−1^ was found for slow growth at 0.017 h^−1^ as well as for the second phase of fedbatches 3 and 4 (*μ* of 0.049 and 0.067 h^−1^) and for fast growth (*μ* of 0.094 and 0.115 h^−1^; Fig. 4, Table 1). Thus, substantial basal levels of >0.4 mg g^−1^ h^−1^ CALB were secreted independent of *μ* ranging from 0.017 to 0.115 h^−1^. Stable *q*_p_ values higher than this basal level were determined during fedbatch cultivations at *μ* around 0.038 h^−1^, corresponding to 25% of *μ*_max_. Temporarily higher *q*_p_ values than this basal level were determined during fedbatch cultivations at higher specific growth rates from 0.049 to 0.067 h^−1^.

To compare results from the strain investigated in this study with data published in the literature, secretion rates were also calculated as protein concentrations correlated with enzyme activity (Fig. 4b). For the fedbatch cultivations, CALB enzyme activity increased with protein concentration in the supernatant. However, the correlation was better for cultivations of short duration where lower protein concentrations were achieved. The specific CALB activity, determined as an average of all fedbatch cultivations with exponential glycerol addition, was 19.3 ± 0.8 U mg^−1^ protein. More than 98% of total protein secreted during the production phases was CALB (determined for fedbatch 1 and fedbatch 2).

#### Design of *μ* shift experiments in fedbatch cultures

The relationship between product formation and specific growth rate is the basis for a rational design for a fedbatch production process. To establish the desired *q*_p_ (*μ*) relationship, a single fedbatch was performed instead of numerous laborious fedbatch cultivations at each preset *μ* value. In the later novel approach, *μ* was varied dynamically within one experiment (dynamic approach), while for the established (second) approach, a constant specific growth rate was maintained during the entire fedbatch experiment.

**Fig. 5:**
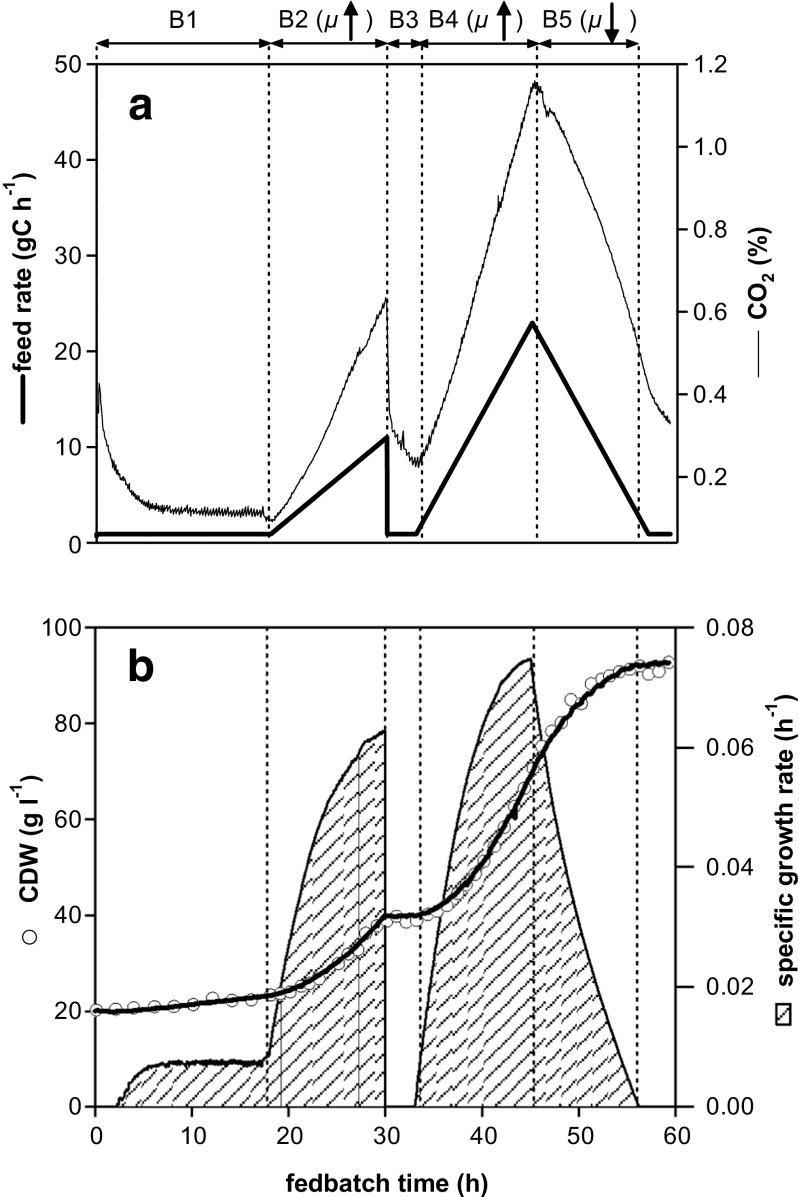
Design of fedbatch to experimentally perform *μ* shifts. The feed profile (graph **a**) and biomass growth (graph **b**) are shown for one selected *μ* shift experiment. After initial batch cultivation (phase A, not shown), the rate of glycerol addition was varied systematically during fedbatch (several B phases). Phases with changing specific growth rates (B2, B4, B5) to investigate culture performance at different *μ* (i.e. screening for *q*_p_) and adaptation phases (B1, B3) are distinguished. Offline measured values are given as *symbols*. The time course of specific growth rate (graph **b**, *dashed area*) is derived from the biomass concentration, which was computed by carbon balancing (graph **b**, *black line*)

In a single fedbatch experiment, by systematically varying the specific growth rate, the production range below 50% of *μ*_max_ was repeatedly tested (Fig. 5). Continuously added glycerol was utilised immediately, and thus, the residual glycerol concentration was ≤1 g l^−1^ (for all phases B1–B5). Growth was therefore controlled by the rate of addition of glycerol (which corresponds to *q*_s_) during the entire fedbatch cultivation. During the initial adaptation phase of 18 h (B1) and an intermediate phase of 4 h (B3), the substrate addition rate was maintained at a constant level (Fig. 5a). During the following ‘diagnostic’ phases (each 12 h, Fig. 5a), the substrate addition rate was increased (phases B2 and B4) and then linearly decreased (phase B5). By linearly increasing or decreasing the glycerol feed addition rate, specific growth rates were dynamically shifted during these periods while ageing cells accumulated in the bioreactor vessel and the biomass concentrations increased (Fig. 5b). Due to the availability of substrate, linear feed profiles resulted in specific growth rates changing in a sigmoidal manner. Biomass concentration therefore increased steadily, but specific growth rate changed between 0.004 and 0.074 h^−1^. During phases B1, B2 and B4, the specific growth rate increased, whereas it decreased during phases B3 and B5. The dynamic *μ* shifts are also depicted in online signals of carbon dioxide in the off gas (Fig. 5a). Using this set-up, several different *μ* values were investigated during a single experiment.

**Fig. 6:**
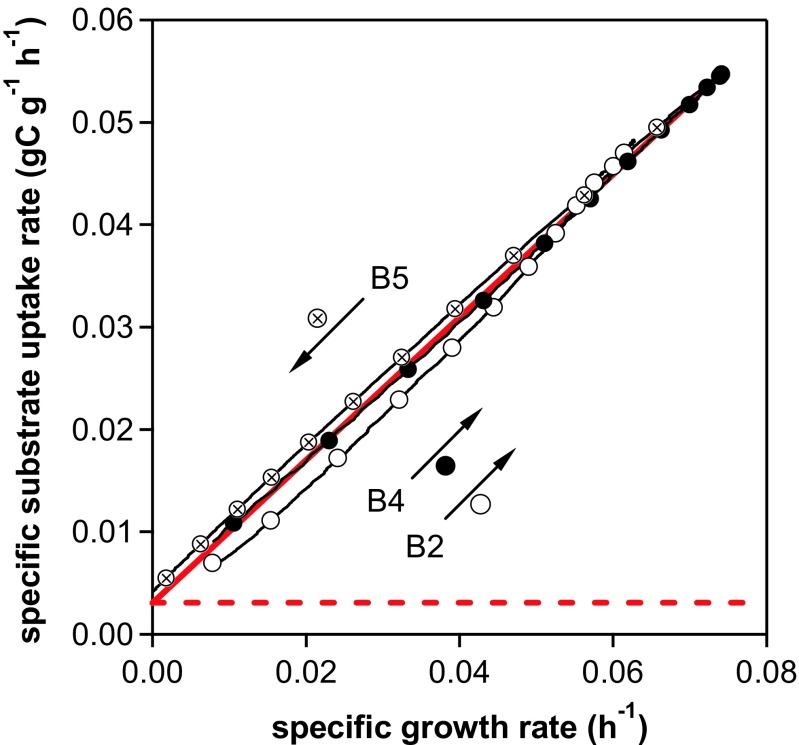
Characterisation of growth by applying dynamic changes to the specific growth rate. For time courses determined at each characterisation phase (B2, B4 and B5) of the *μ* shift experiment (Fig. 5b), values of specific substrate uptake rate are plotted against corresponding pair values of specific growth rate. The values were derived either from offline data (*circles*) or from biomass concentration computed by online carbon balancing (*lines*). The direction of *μ* change during each phase is indicated by an *arrow*, phase designation and the corresponding *symbol*. By linear regression, the mean specific maintenance rate (*m*_s_) was determined from offline values as a *y*-axis intercept of (0.0031 ± 0.0008) gC g^−1^ h^−1^ and is indicated by the *dashed line*

The influence of increasing biomass concentration and cultivation time (or cell age) on biomass growth was tested by establishing the *q*_s_(*μ*) relationship for the three *μ* shift phases from dynamic fedbatch cultivation data (raw data given in Fig. 5). For all three phases (B2, B4, B5), the specific growth rate closely followed the increase and decrease in specific substrate uptake (Fig. 6). The first *μ* shift phase, B2, showed lower initial substrate uptake than the following phases, B4 and B5. The substrate uptake without biomass growth (i.e. the intercept on the *y*-axis) corresponds to the specific substrate uptake rate for cell maintenance (*m*_s_) in gC g^−1^ h^−1^ (Fig. 6). During the three constitutive shifting phases B2, B4 and B5, *m*_s_ values increased by 0.0019 ± 0.0001, 0.0029 ± 0.0001 and 0.0047 ± 0.0001 gC g^−1^ h^−1^, respectively. The specific maintenance rate was about 2.5 times higher for the last phase, B5, than for the first phase (i.e. all computations based on highly frequent online data). Based on offline data, the *m*_s_ values for the different phases were not significantly different (within the 95% confidence intervals). The mean specific substrate uptake rate for cell maintenance computed from offline values of all three phases was 0.0031 ± 0.0008 gC g^−1^ h^−1^.

#### Characterisation of product secretion with *μ* shift experiments

The potential of the dynamic approach to replace the established labour-intensive strain characterisation and to speed up the determination of the *q*_p_(*μ*) relationship was evaluated in three identical *μ* shift experiments using glycerol as the sole carbon and energy source.

**Fig. 7:**
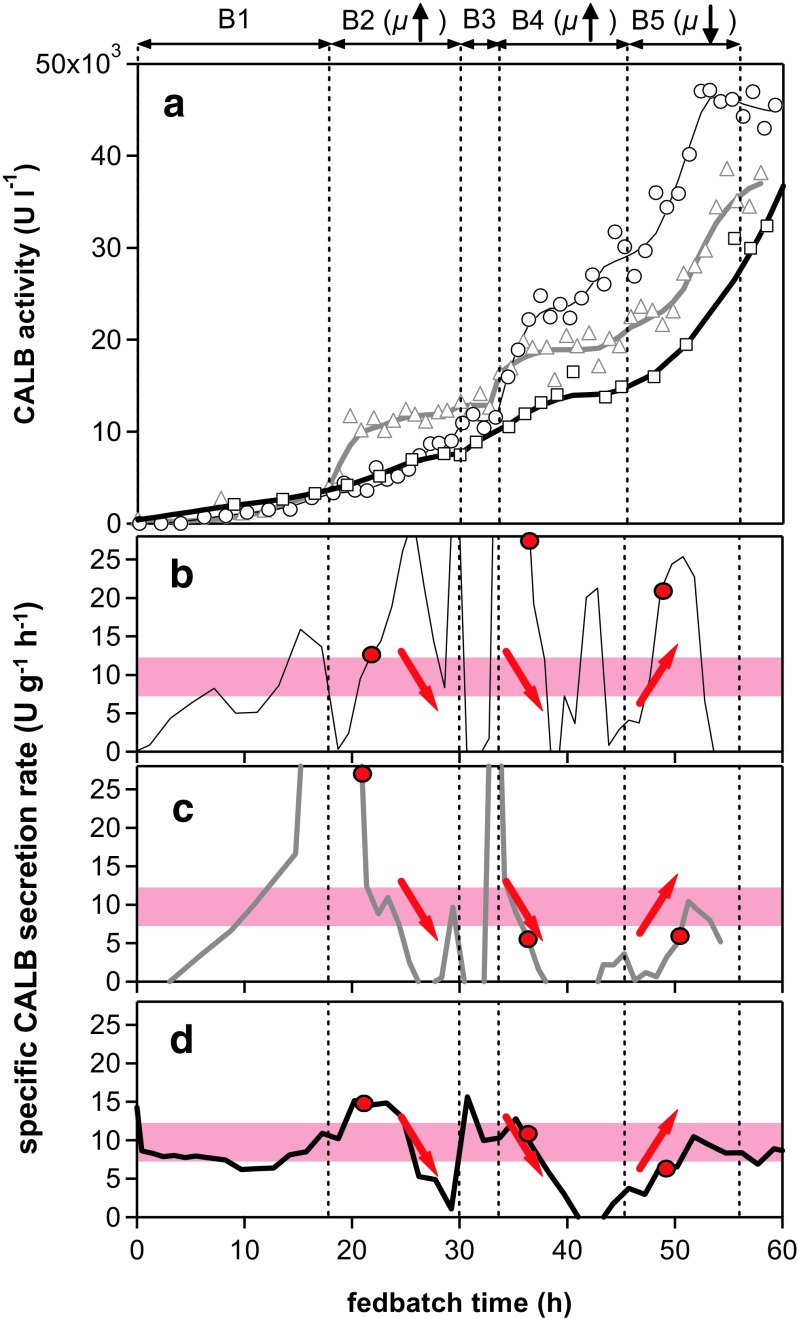
Strain and promoter characterisation with a *μ* shift experiment. Product concentration (graph **a**) and specific product formation rate (graphs **b**–**d**) are shown for three *μ* shift experiments with the same set-up as shown in Fig. 5. Specific growth rate was increased during screening phases B2 and B4 and decreased during phase B5. CALB activities are shown as *symbols* (graph **a**). Time courses of product activity (shown as *lines* in graph **a**) were smoothed by a moving average algorithm. Calculated specific product secretion rates are shown in graphs **b**–**d**. Graph **b** corresponds to *open circles* and a thin *black line* of graph **a** (and data shown in Fig. 5). Graph **c** corresponds to *open triangles* and a *thick grey line*. Graph **d** corresponds to *open squares* and a *thick black line*. General trends of the changes of *q*_p_ are indicated by *thick arrows* in graphs **b**–**d**. Specific secretion rates of 7.4 to 12.1 U g^−1^ h^−1^, which correspond to 0.4 to 0.6 mg g^−1^ h^−1^ (basal level defined in Fig 4a), are indicated by the *shaded area*, and the *y*-axis is scaled according to the *y*-axis of Fig 4a. *q*_p_ at the optimum specific growth rate of 0.038 h^−1^, at which the maximum specific product formation was achieved (*vertical line* in Fig 4a), is indicated as *circles*.

Cell growth closely followed dynamic changes in the added amount of substrate per gram of biomass. The amount of secreted product (CALB) was also affected by dynamic changes in growth conditions (Fig. 7). The repeated changes in substrate availability in one single cultivation showed an effect on the amount of product secreted (CALB activity in the supernatant). For three different *μ* shift experiments with the same experimental set-up, the *q*_p_ data were poorly reproducible in absolute numerical values.

A comparable pattern of changes in product secretion was, however, observed in all three *μ* shift cultures: a decreasing secretion towards the end of phase B2 and during phase B4 as well as an increasing secretion at the beginning of phase B5 (Fig. 7a). These common changes in all three *μ* shift experiments were also reflected in the time courses of specific product secretion rates (Fig. 7b–d) and are indicated by arrows. A decrease in the specific secretion rate was observed for all three experiments towards the end of phase B2 at high specific growth rates. Measured CALB activity stagnated, and the derived *q*_p_ values decreased before substrate addition was abruptly reduced at the beginning of phase B3. At the end of the intermediate phase B3 and at the beginning of increased substrate addition (and *μ*) during phase B4, secreted product increased substantially but soon (after about 3 h) increased again for all three experiments. Phase B4, with an increasing specific growth rate, was a repetition of phase B2, for which a decrease in specific product secretion was also observed. Interestingly, after continuously decreasing the feed rate at the beginning of the last phase, B5, all three experiments again showed steeply increasing product activities and consequently increasing specific product secretion rates.

The measured concentrations/activities of secreted product and time courses were different for the individual experiments (each at a constant *μ*), but the common trends in changing specific secretion rates, as triggered by changing the feed rate and the specific growth rate, were reproducible; a decrease in feed rate (and thus the specific growth rate) showed the opposite effect on *q*_p_ as an increase in feed rates during phases B2 and B4. Although secretion of product was affected by the dynamic changes in growth conditions, the time courses of *q*_p_ were highly sensitive, allowing a reproducible *q*_p_(*μ*) relationship to be established from different experiments. A high proportion of *q*_p_ values, as determined by the dynamic approach, were comparable to basal levels determined elsewhere (shaded area in Figs. 4a and 7). In contrast to single fedbatch cultivation with exponential feed addition, where the specific secretion rate is determined as an average over a distinctly longer time period of several hours, in the dynamic experiments, *q*_p_(*t*) reflects immediate changes (Fig. 7b), which were transiently higher or lower than the basal *q*_p_ level.

The dynamic characterisation of the *q*_p_(*μ*) relationship tested here is different from the approach using several single fedbatch cultivations at constant specific growth rates. The relationship between specific product formation rate and *μ* was reproducible as *q*_p_ was inversely related to *μ*. A rough estimation of a suitable *μ* range for optimum production is therefore possible with significantly reduced experimental effort.

## Discussion

Several promoters for methanol-free production of recombinant protein by *P. pastoris* have recently been developed, and the general principle of regulation by repression/derepression with glucose or glycerol as the sole substrate has been investigated (Capone et al. 2015; Hartner et al. 2008; Mellitzer et al. 2014; Prielhofer et al. 2013; Ruth et al. 2010; Vogl et al. 2016). However, little is known beyond the dynamic conditions for switching on and off in order to control these modified promoters using appropriate process strategies. A new P_AOX1_ variant, which is a variation of the d6 promoter described by Hartner et al. (2008), was, for the first time, characterised comprehensively in this study.

### Relationship between CALB production controlled by the P_AOX1_ variant and specific growth rate

For the P_AOX1_ variant, complete repression of product formation was achieved during batch cultivation with excess glycerol, where glycerol concentrations remained above 10 g l^−1^ and CALB activity was detectable neither in the supernatant nor intracellularly. The repression/derepression principle proposed by Egli et al. (1980) for native alcohol oxidase activity in *Hansenula polymorpha* cells grown with glucose is related to specific growth rate. In this study, the relationship between specific CALB secretion rate and specific growth rate was established for *P. pastoris* growing with glycerol under limited (≪1 g l^−1^) substrate availability in fedbatch culture and, thus, with gene expression controlled by a new synthetic P_AOX1_ variant. This *q*_p_(*μ*) relationship reflects the equilibrium between various intracellular processes until the product is secreted (i.e. induction of gene expression, translation, protein folding, translocation, potential degradation in the endoplasmic reticulum, flux of folded protein out of the ER and trafficking through the secretory machinery).

Maximum constant specific productivity of 0.93 ± 0.06 mg g^−1^ h^−1^ was achieved at 25% of *μ*_max_ or at *μ* values around 0.038 h^−1^. Maximum specific secretion rates at *μ* considerably below *μ*_max_ were often found for high-level protein secretion controlled by strong P_AOX1_ promoters induced by methanol (Kobayashi et al. 2000; Potgieter et al. 2010; Wu et al. 2010), while the opposite was found for P_GAP_-controlled product formation (Looser et al. 2015). Specific rates of P_GAP_-controlled protein secretion with glucose as substrate typically increase with a specific growth rate close to *μ*_max_ (Buchetics et al. 2011; Maurer et al. 2006; Rebnegger et al. 2014). Secretion was found to be coupled to specific growth rate and cell cycle for P_GAP_-regulated expression; for P_GAP_-regulated expression, secretion was enhanced at higher specific growth rates, and at higher *μ*, a larger fraction of cells were in the G2 and M phases of the cell cycle (Buchetics et al. 2011). The main differences between the two systems (P_AOX1_ or P_GAP_) were the substrates (methanol or glucose) and strain design (i.e. inducible or constitutive promoter).

The same promoter and substrate combination tended to have a greater influence on the product formation kinetics than similar categories of recombinant products (Looser et al. 2015). Recent analysis of the secretory capacity of *P. pastoris* at the single cell level indicated that increasing gene copy number under the control of the P_AOX1_ promoter decreased the rate of secretion for proteins of various complexities, while an increase in copy number did not seem to saturate the secretory capacity of the strain using P_GAP_ (Love et al. 2012).

Moreover, a comparative study with 48 different codon-optimised synthetic genes coding for the same protein sequence and under control of four different promoters (P_GAP_, P_AOX1_ and two other synthetic P_AOX1_ variants: P_En_ with enhanced production with methanol and P_Des_ with strong transcription due to derepression without induction by methanol) showed that a high gene dosage negatively influenced protein production (during fedbatch cultivation) (Mellitzer et al. 2014). Several authors proposed an overload of the secretory pathway of the cells due to high transcript levels, which can result from strong promoters, optimised gene sequences or high gene dosages (Glick 1995; Hohenblum et al. 2004; Love et al. 2012; Mellitzer et al. 2014). Furthermore, for heterologous gene expression under control of a synthetic P_AOX1_ variant (P_Des_) and using glycerol as substrate, a negative effect on protein production was observed if the gene dosage was too high (>4–9 gene copies) (Mellitzer et al. 2014). However, using the same P_Des_ promoter variant, this effect was more pronounced with methanol as substrate than with glycerol (Mellitzer et al. 2014).

The *P. pastoris* strain used in the present study contained two gene copies of *calB* and was grown on glycerol. To improve folding capacity, an optimised gene coding for protein disulphide isomerase was expressed under the control of the same promoter as *calB.* By achieving stable specific secretion rates of around >0.4 mg g^−1^ h^−1^, this *P. pastoris* strain reached high *q*_p_ values. Specific secretion rates of recombinant proteins are often below 0.4 mg g^−1^ h^−1^ for *P. pastoris* using P_AOX1_ or P_GAP_ (Barrigon et al. 2015; Looser et al. 2015). For instance, published specific secretion rates (recalculated in mg product built per g cell dry weight and per hour) are 0.17 mg g^−1^ h^−1^ with a glycoengineered *P. pastoris* strain producing recombinant IgG1 antibody (P_AOX1_, methanol, *μ =* 0.014 h^−1^, Potgieter et al. 2010), 0.26 mg g^−1^ h^−1^ recombinant human serum albumin (P_AOX1_, methanol, *μ =* 0.002 h^−1^, Kobayashi et al. 2000), 0.42 mg g^−1^ h^−1^ recombinant α-galactosidase (P_AOX1_, methanol, *μ =* 0.03 h^−1^, Zhang et al. 2005) and 0.69 mg g^−1^ h^−1^ recombinant porcine trypsinogen (P_AOX1_, substrate mixture methanol-glucose, *μ =* 0.07 h^−1^, Paulova et al. 2012).

For the strain in this study, 0.4 to 0.6 mg g^−1^ h^−1^ represented a basal level of CALB secretion, which was independent of the specific growth rate applied (Fig. 4). However, at specific growth rates of 0.049 and 0.067 h^−1^, considerably higher specific production rates between 1.03 and 1.15 mg g^−1^ h^−1^ were observed in about the first 20 h of fedbatch cultivation. Afterwards, specific secretion rates decreased by 57 to 65% to a basal level of 0.40 to 0.44 mg g^−1^ h^−1^ (Table 1). These transiently high but unstable specific secretion rates can be interpreted as an indication of limited production or secretory capacity with increasing cultivation time and at higher specific growth rates. It can be assumed that strong transcription and therefore an excess protein load in the secretory pathway may result in inefficient recycling by the protein export machinery (Love et al. 2012), which is more pronounced at high specific growth rates than at lower ones. In addition, incorrect folding of the recombinant human consensus interferon mutant (intracellularly and secreted) at higher specific growth rates (0.027 h^−1^) with methanol, under the control of P_AOX1_, was reported, while product was correctly formed at low *μ* = 0.01 h^−1^ (Wu et al. 2010).

Our investigation of different specific growth rates for the P_AOX1_ variant suggests that the secretion kinetics observed are not dominated by promoter regulation but instead by production/secretion capacity. The maximum product formation at *μ* considerably below *μ*_max_ (25% of *μ*_max_), shown here with the P_AOX1_ variant, is more closely related to P_AOX1_-controlled secretion kinetics with methanol than P_GAP_-controlled product formation with glucose. High product formation at low specific growth rates is favourable to the design of fedbatch production processes for high productivity (Buchetics et al. 2011). In general, high productivity and a high final titre are reached if cultivation occurs at a high biomass concentration, for long periods, and at a desired *μ* for product formation before bioreactor system boundaries are reached (Looser et al. 2015).

### Characterisation of product secretion with a dynamic fedbatch approach (*μ* shift)

The dynamic fedbatch cultivations trialled in this study revealed information on the dynamic effects of changes in specific growth rates and substrate availability on product secretion. Interestingly, after continuously decreasing the feed rate at the beginning of the last phase, B5 (Fig. 7), all three experiments showed steeply increasing product activities and increasing specific product secretion rates. The continuous reduction in feed rate and specific growth rate had the opposite effect as continuously increasing feed rate and specific growth rate during phases B2 and B4. Although absolute reproducibility was low for three single *μ* shift experiments with the same set-up, comparable patterns of changes in product secretion were observed for all three dynamic experiments (Fig. 7).

A transient response that is unique to each strain has been observed for initial adaptation to methanol utilisation (Dietzsch et al. 2011b; Hesketh et al. 2013). Independent of the actual production of recombinant protein, stress responses (including unfolded protein response) were found to be induced within 3 h after methanol was pulsed into a continuous culture with sorbitol (Hesketh et al. 2013). According to Hesketh et al. (2013), the cells no longer exhibited stress at a transcriptional level once steady-state growth had been established on the new substrate combination (methanol/sorbitol), although they continued to produce the recombinant protein. Adaptation to steady-state methanol utilisation seems to be ‘more stressful’ at a transcriptional level than recombinant protein production itself. This kind of strain-specific response is attributed to the switch from a non-inducing substrate (sorbitol, glucose or glycerol) to the inducing substrate, methanol. However, in the dynamic approach of this study, glycerol was the only substrate applied, but substrate addition rate (and therefore specific growth rate and cell physiology) changed continuously.

Oscillating feeding has been described as having a positive effect on productivity of P_AOX1_-controlled product formation (Dietzsch et al. 2011a; Spadiut et al. 2013). A stepwise increase in the feed rate was described as more beneficial than linearly increasing specific substrate addition ramps (Spadiut et al. 2014). Spadiut and Herwig (2014) speculated that cells may secrete more product when they are challenged by a stepwise increase in feed rate, but after each increase, they still have time to adapt to culture conditions. Time to adapt, however, is difficult to specify. Signalling, according to Almquist et al. (2014), occurs over the timescale range of 0.001 to 1 s, gene expression over a timescale of 1 to 500 s, protein secretion over the range of 8 to 167 min and the cell cycle over the timescale of 0.3 to 28 h. Changes in specific growth rate during dynamic fedbatch cultivation between 0.004 and 0.074 h^−1^ corresponded to an average cell cycle time (doubling time) of 173.3 to 9.4 h. Hence, during each characterisation phase of 12 h (Figs. 5 and 7, B2, B4, B5), the doubling time constantly changed from 173.3 to 9.4 h (for average biomass). In respect of this change, a high sampling rate and/or online measurements were crucial to evaluate and interpret the impact of constantly changing substrate availability and specific growth rate on cell physiology and secretion of recombinant product.

Although the desired *q*_p_(*μ*) relationship could not be accurately established by dynamic variations in specific growth rate within a single fedbatch experiment, a rough estimation of suitable *μ* ranges for optimised production was achieved in accordance with the *q*_p_(*μ*) relationship established from several exponential fedbatch cultivations. The maximum stable specific product secretion rate was found at low specific growth rates ≤0.038 h^−1^, corresponding to ≤25% of *μ*_max_. High product formation at low specific growth rates is favourable to the design of high productivity fedbatch processes since product formation can take place at a desired *μ*, for a long period at a high biomass concentration before system boundaries are reached. To enable new P_AOX1_ variants to reach maximum titres, future studies will focus on refinement of the model by investigating optimum production strategies.

### Which approach is more time-saving and informative?

With respect to time-to-the market, biotechnological processes are still outcompeted by less environmental friendly extraction of animal or plant material or by chemical synthesis. Systematic improvements in methods for biotechnological process development are therefore crucial.

The establishment of a comprehensive *q*_p_(*μ*) relationship for a new strain typically comprises several experiments at different pre-set *μ* values and would require five or more times the experimental effort of a *μ* shift experiment, in which *μ* is changed over its entire range (Fig. 5). The use of dynamic process conditions for rapid physiological strain characterisation is described by Spadiut et al. (2013) and Spadiut and Herwig (2014). The different dynamic fedbatch approaches presently being pursued could enable the entire production range of *q*_s_ (and *μ*) to be investigated in one experiment (Capone et al. 2015; Spadiut et al. 2014; Zalai et al. 2012).

Though less accurate, the information gained in such a single *μ* shift experiment is valuable as the first estimate of appropriate conditions with respect to optimum specific growth rate for maximum production (*μ*_opt_). In particular, *μ*_opt_ can be derived from *q*_p_(*t*) maxima (Fig. 7b–d), and an understanding of system dynamics under changing *μ* can be gained. Though highly relevant, the latter information on system dynamics, which reflects increased production capability (Spadiut and Herwig, 2014), would be lost during a conventional fedbatch experiment at constant *μ* or in continuous (chemostat) cultures typically used to establish the *q*_p_(*μ*) relationship. By applying the *μ* shift approach described here, a new paradigm for rational bioprocess development was established: Process complexity and raw data evaluation increase, but the experimental load in the laboratory decreases.

The new derepressible P_AOX1_ variant allows high specific productivity of a secreted heterologous protein like CALB in *P. pastoris* without methanol induction. During comprehensive characterisation of the new promoter, phases of stable and reproducible productivities were identified, and varying secretion levels were observed and described. Recombinant protein was generally not produced when there was an excess of substrate, and appropriate process control strategies combining phases of biomass growth and protein production are therefore feasible. Interestingly, basal secretion levels were observed during substrate-limited growth, independent of varying specific growth rates.

These new findings are highly significant since knowledge of production kinetics is a prerequisite for production process design, where the use of methanol (and the conventional AOX1 promoter) may be problematic. Further information on promoter control by appropriate cultivation strategies will advance *P. pastoris* production systems in the future.
